# Analysis of Extreme Phenotype Bulk Copy Number Variation (XP-CNV) Identified the Association of *rp1* with Resistance to Goss's Wilt of Maize

**DOI:** 10.3389/fpls.2018.00110

**Published:** 2018-02-09

**Authors:** Ying Hu, Jie Ren, Zhao Peng, Arnoldo A. Umana, Ha Le, Tatiana Danilova, Junjie Fu, Haiyan Wang, Alison Robertson, Scot H. Hulbert, Frank F. White, Sanzhen Liu

**Affiliations:** ^1^Department of Plant Pathology, Kansas State University, Manhattan, KS, United States; ^2^Department of Plant Pathology, University of Florida, Gainesville, FL, United States; ^3^Institute of Crop Science, Chinese Academy of Agricultural Sciences, Beijing, China; ^4^Department of Statistics, Kansas State University, Manhattan, KS, United States; ^5^Department of Plant Pathology and Microbiology, Iowa State University, Ames, IA, United States; ^6^Department of Plant Pathology, Washington State University, Pullman, WA, United States

**Keywords:** goss's wilt, resistance, copy number variation, *rp1*, *Zea mays*

## Abstract

Goss's wilt (GW) of maize is caused by the Gram-positive bacterium *Clavibacter michiganensis* subsp. *nebraskensis* (Cmn) and has spread in recent years throughout the Great Plains, posing a threat to production. The genetic basis of plant resistance is unknown. Here, a simple method for quantifying disease symptoms was developed and used to select cohorts of highly resistant and highly susceptible lines known as extreme phenotypes (XP). Copy number variation (CNV) analyses using whole genome sequences of bulked XP revealed 141 genes containing CNV between the two XP groups. The CNV genes include the previously identified common rust resistant locus *rp1*. Multiple *Rp1* accessions with distinct *rp1* haplotypes in an otherwise susceptible accession exhibited hypersensitive responses upon inoculation. GW provides an excellent system for the genetic dissection of diseases caused by closely related subspecies of *C. michiganesis*. Further work will facilitate breeding strategies to control GW and provide needed insight into the resistance mechanism of important related diseases such as bacterial canker of tomato and bacterial ring rot of potato.

## Introduction

Goss's bacterial wilt and leaf blight of maize, or simply Goss's wilt (GW), was first identified in 1969 (Schuster et al., 1972; Ruhl et al., 2009). In recent years, the disease has emerged as a serious threat to production (Jackson et al., 2007; Wise et al., 2009; Mueller and Wise, 2014). The estimated total yield loss due to GW, from 2012 to 2015, was more than 500 million bushels in the US and Canada (Mueller et al., 2016). *Clavibacter michiganensis* subsp. *nebraskensis* (Cmn), the causal agent of GW, is a Gram-positive bacterium (Phylum Actinobacteria) and a vascular pathogen of maize, causing systemic wilting on young plants and leaf blight at all stages of plant growth (Ruhl et al., 2009). However, the genetic basis of host resistance to actinobacteria, in general, is poorly understood. The lack of genetic resources to control *C. michiganesis*, specifically, has led to extensive quarantine measures, particularly, for ring spot of potato, which is caused by *C. michiganesis* subsp. *sepedonicus*. Quarantine measures are also targeted to *C. michiganesis* subsp. *michiganesis*, a related wilt and canker pathogen of tomato. Ectopic expression of genes conferring broad-spectrum antimicrobial activities was reported to enhance basal defenses of tomato against a related wilt pathogen *C. michiganensis* subsp. *michiganensis* (Kabelka et al., 2002; Coaker and Francis, 2004; Balaji and Smart, 2012). Two additional subspecies, *C. michiganesis* subsp. *tesselarius* and *C. michiganesis* subsp. *insidiosus*, cause diseases of wheat and alfalfa, respectively (Francis et al., 2010). A sixth subspecies has been recently proposed, *C. michiganesis* subsp. *phaseoli*, which causes a systemic disease in common bean (González and Trapiello, 2014).

Plant defense genes dynamically co-evolve with diverse pathogens, and are, therefore, highly polymorphic (Brooks et al., 2006). In general, the adaptive value of defense genes may impose fitness costs and, consequently, they may be eliminated without compromising plant health (Tian et al., 2003). Pathogen resistant (R) genes can occur in clusters at specific genomic loci, particularly in cases of common R gene classes (Marone et al., 2013). Genes in clusters may have adaptive advantages derived from rapid evolution due to rearrangement. Rearrangement can generate new specificities, and under low disease pressure, eliminate deleterious genes, thus generating intra-species copy number variation (CNV) that includes presence and absence variation (PAV). CNV has been identified for plant disease defense genes in a range of species (Bakker et al., 2006; Shen et al., 2006; Cook et al., 2012; Xu et al., 2012; González et al., 2013; Golicz et al., 2016). For example, the 31 kb locus *Rhg1* confers resistance to soybean cyst nematode and appears to function due to multiplication of the locus (Cook et al., 2012).

Maize genomes exhibit high levels of genomic structural variation (Schnable et al., 2009; Springer et al., 2009; Beló et al., 2010; Hirsch et al., 2014). Analysis of sequences of 27 diverse maize inbred lines indicated that the B73 reference genome contains ~70% low copy sequences (Gore et al., 2009). Transcriptomic analysis of 503 diverse maize inbred lines identified 8,681 representative transcript assemblies that are absent in the B73 reference genome (Hirsch et al., 2014). Furthermore, genotyping-by-sequencing (GBS) analysis of 14,129 maize inbred lines found 1.1 million PAVs (Lu et al., 2015a). Relative to inbred line B73, genes with reduced copy number in non-B73 lines are enriched in the pathways of stress responses, indicative of high variability in copy number for disease defense genes (Swanson-Wagner et al., 2010). For example, the *rp1* locus is a highly variable genomic complex of maize, conferring race-specific resistance to the common rust fungus (Hulbert, 1997; Smith et al., 2004). The locus carries multiple *rp1* paralogs, which are members of the nucleotide binding site leucine-rich repeat (NLR) family. Unequal crossovers generate gene duplications, gene losses, and genes with new phenotypic characteristics, and, ultimately, yielding lines with diverse haplotypes at the *rp1* region (Bennetzen et al., 1988; Richter et al., 1995). The number of *rp1* paralogs varies in a broad range from one to greater than fifty copies (Smith et al., 2004). A similar example of a resistant gene that varies in copy number is the recently cloned maize wall-associated kinase (ZmWAK) that confers resistance to head smut. ZmWAK is absent in many modern maize lines, while present in wild relatives. Absence of ZmWAK is highly correlated with high susceptibility to head smut (Zuo et al., 2015). Despite the few examples, the functional consequences of CNV to plant defense are largely unexplored, and the methodology for such studies needs improvements. Here, we established a simple phenotyping method to quantify disease symptoms and identified disease defense associated genes through comparing average copy number between bulked pools of individual maize lines that displayed highly resistant or susceptible phenotypes, which was referred to as extreme phenotype bulk copy number variation (XP-CNV) analysis.

## Materials and methods

### Genetic materials

Six hundred and fifteen maize accessions (largely inbred lines) that were subjected to disease phenotyping, including lines from the Maize 282 Association Panel (Flint-Garcia et al., 2005), were ordered from North Central Regional Plant Introduction Station (NCRPIS). These accessions were used to identify GW resistant and susceptible lines. To validate the association between the *rp1* locus and GW resistance, *Rp1, Rp3*, and *Rp5* accessions were used. These accessions were maintained and introgressed to the GW susceptible inbred line H95.

### Quantitative phenotyping of disease symptoms

A method was established for quantification of GW disease development. A virulence strain CMN06-1, isolated from Iowa maize field in 2006 by Dr. Charlie Block, was cultured on nutrient broth yeast extraction (NBY) medium at 28°C for 2–3 days. Maize plants were grown in the greenhouse at 28°C with a 14 h photoperiod. The third leaf of three-leaf maize seedlings was inoculated by cutting at 2 cm from the tip with scissors dipped in bacterial inoculum of optical density of 0.55–0.60 at 600 nm. Lengths of lesion were measured from the cut surface at the tip to the distal-most position on the leaf that exhibits a gray, chlorotic or water-soaked lesion at 13 days post-inoculation (DPI). Common lines were used in different batches to examine batch effects.

### Selection of highly resistant (R) lines and highly susceptible (S) lines

The lesion phenotyping protocol was used to inoculate the third leaf of three-leaf seedlings. An exception was made for seedlings (335 out of 2,958) that had not developed to the desirable stage on the inoculation day. For those cases, the second or fourth leaves were inoculated instead. From phenotyping data, a statistically significant but weak association was found between seedling heights, from soil surface to the top node of plants, and lesion lengths. To generate comparable lesion phenotypes among maize genotypes, a linear model to obtain best linear unbiased estimation (BLUE) was applied. The model used raw lesion lengths measured at 13 DPI and genotype as response variable and explanatory variable, respectively. Seedling height, inoculation leaf, and batch were used as other covariates in the model. The BLUE values of lesion lengths were used to represent host resistance levels. Maize genotypes with estimated lesions less than 9 cm were considered as highly resistant (R) genotypes, while those with lesions higher than 22 cm or those with lesions higher than 20 cm and 80% inoculation leaves showing whole leaf wilting were considered as highly susceptible (S) genotypes. Based on genetic relations inferred from genotypes, some highly R and S lines were removed to reduce the genetic redundancies from closely related lines. Selected highly R (*N* = 37) or highly S lines (*N* = 44) were used for the XP-CNV analysis.

### WGS sequencing of highly R and S pools

Seeds were germinated and grown in the greenhouse at 28°C with a 14 h photoperiod. Fresh leaves of seedlings at the 2–3 leaf-stage were harvested. Tissues of highly R lines and highly S lines were pooled to form highly R pool and highly S pool, respectively. Pooled tissues were frozen in liquid nitrogen, and homogenized with liquid nitrogen to fine powder. Nuclei were extracted to reduce the proportion of DNAs from organelle genomes, followed by using the Qiagen DNeasy Plant Mini Kit protocol to extract nucleus DNA (Zhang et al., 2012; Liu et al., 2017). Nucleus genomic DNAs were used for TruSeq PCR-free library preparation. Two biologically replicated samples for each of highly R and highly S pools were prepared and subjected to WGS sequencing with one sample per lane using a HiSeq2500. Paired-end reads (2 × 126 bp) were generated. Sequencing was conducted at Macrogen, Inc., South Korea.

### Trimming and alignment of WGS sequences

The software Trimmomatic (version 0.32) was used to trim adaptor sequences and low quality sequences (Bolger et al., 2014) with the parameter of “ILLUMINACLIP:<adaptor>:3:20:10:1:true LEADING:3 TRAILING:3 SLIDINGWINDOW:4:13 MINLEN:50.” The adaptor sequence was from https://github.com/timflutre/trimmomatic/blob/master/adapters/TruSeq3-PE.fa. Reads retained after trimming were aligned to the B73 reference genome (B73Ref2; Schnable et al., 2009) using BWA (0.7.15-r1140) with the “mem” module (Li and Durbin, 2009). The minimal mapping score of 40 was required and an in-house script (github.com/liu3zhenlab/scripts/blob/master/bwa.filter/samparser.bwa.pl) was used to filter alignments to ensure that reads were uniquely aligned with high confidence. Specifically, each alignment was required to have the insert between 150 and 800 bp, at least 50 bp alignment length, at least 96% identity, and at most 4% unaligned percentage of a read length. The samtools software (version 1.1; Li et al., 2009a) was used to convert alignments in the SAM format to the BAM format.

### Statistical analysis of CNV between R and S pools

HTSeq (0.6.1p1) was used to count read depth for each gene (filtered gene set, 5b) using the “union” mode. The generalized linear model, implemented in the DESeq2 package (version 1.4.5), for read counts of genes with at least 20 total reads from all four samples was used for testing the null hypothesis that no difference in read depth between highly R pools and highly S pools (Love et al., 2014). A false discovery rate (FDR) approach was used to account for multiple tests (Benjamini and Hochberg, 1995). The FDR 10% was used as the cutoff for declaration of significant CNVs.

### Gene annotation

Gene annotation was downloaded from the annotation at Phytozome (https://phytozome.jgi.doe.gov/pz/portal.html#!bulk?org=Org_Zmay) and the B73Ref2 annotation at maizesequence.org.

### GO enrichment test of CNV genes

Enrichment analysis was performed for determining if a certain GO term is over-represented in Up-CNV genes (greater copies in R pools) vs. Dn-CNV genes (fewer copies in R pools), and vice versa. The randomly resampling method (*N* = 10,000) in the GOSeq enrichment test (Young et al., 2010) was employed. GO terms with p-values smaller than 0.05 were considered to be over-represented in a group.

### Validation of CNV genes using WGS sequencing data of the Maize 282 Association Panel

WGS data of the Maize 282 Association Panel were downloaded from iplant shared from the Panzea research group. Among all WGS sequenced lines, 239 maize lines were phenotyped in this study. Sequencing reads were trimmed and aligned to the B73 reference genome (B73Ref2; Schnable et al., 2009) with the same procedures as used for highly R and S WGS data. HTSeq (0.6.1p1) was applied to count read depth for each gene (filtered gene set, 5b) using the “union” mode. Total pairs of reads after trimming were used to determine RPM (read pairs per million of total read pairs) of each gene as normalized values. The correlation was determined between normalized read counts and phenotypic values for each examined gene.

### Quantitative real-time PCR (qPCR)

Genomic DNA was isolated using fresh leaves of seedlings at the 2–3 leaf-stage. qPCR was performed in 10 μL reactions containing 4.2 μL g DNA, 0.4 μL 10 mM of each primer, and 5 μL 2x SYBR Green PCR Master Mix (Bio-Rad) on the CFX96™ real time system (Bio-Rad). Primer efficiencies were measured and relative copy number was calculated using the comparative Ct method (Livak and Schmittgen, 2001). The *actin* gene was used as the endogenous control. A gene specific primer pair was designed to amplify GRMZM2G005134. Primers for qPCR were listed in the Supplementary Table 1.

### Cytogenetic analysis

Maize somatic chromosome preparations using the drop technique, direct probe labeling by nick translation and the florescence *in situ* hybridization (FISH) were performed as described previously (Kato et al., 2004, 2006). The clone containing ~1.7 kb part of *rp1* gene was provided by Dr. James A. Birchler. The insert was sequenced by Sanger sequencing and found 100% similar to the first 1,521 bp of the coding region of the *Rp1-D* allele (Genbank accessions: AY581258 and AF107293) (Supplementary Figure 1), corresponding to the N-terminal NBS region of the *rp1* gene, which is the most conservative part of the gene (Ramakrishna et al., 2002). To make the FISH probe, the insert was amplified with standard primers M13; PCR products were purified with Invitrogen PCR purification kit (Life Technologies, USA) and labeled with Texas red-5-dCTP (PerkinElmer, USA). Oligonucleotide probes labeling centromeric repeats CentC CCTAAAGTAGTGGATTGGGCATGTTCG and 5S ribosomal DNA TAGTAAAAATGGGTGACCGTTCTCGTGTTA were synthesized by Integrated DNA Technologies with 6-FAM attached to the 5′ end. For nucleolus organizing region (NOR) probe, wheat clone pTa71 (Gerlach and Bedbrook, 1979) was labeled with Fluorescein-12-dUTP (PerkinElmer, USA). Chromosome preparations were mounted and counterstained with 4',6-diamidino-2-phenylindole solution (DAPI) in Vectashield (Vector Laboratories, USA). Images were captured with a Zeiss Axioplan 2 microscope using a cooled charge-coupled device camera CoolSNAP HQ2 (Photometrics, USA) and AxioVision 4.8 software (Zeiss). Images were processed using the Adobe Photoshop software.

### Data access

WGS Illumina sequencing data of the pools of highly R and S lines have been deposited at Sequence Read Archive (SRP100278).

## Results

### A simple inoculation method for GW disease quantification

**Figure 1:**
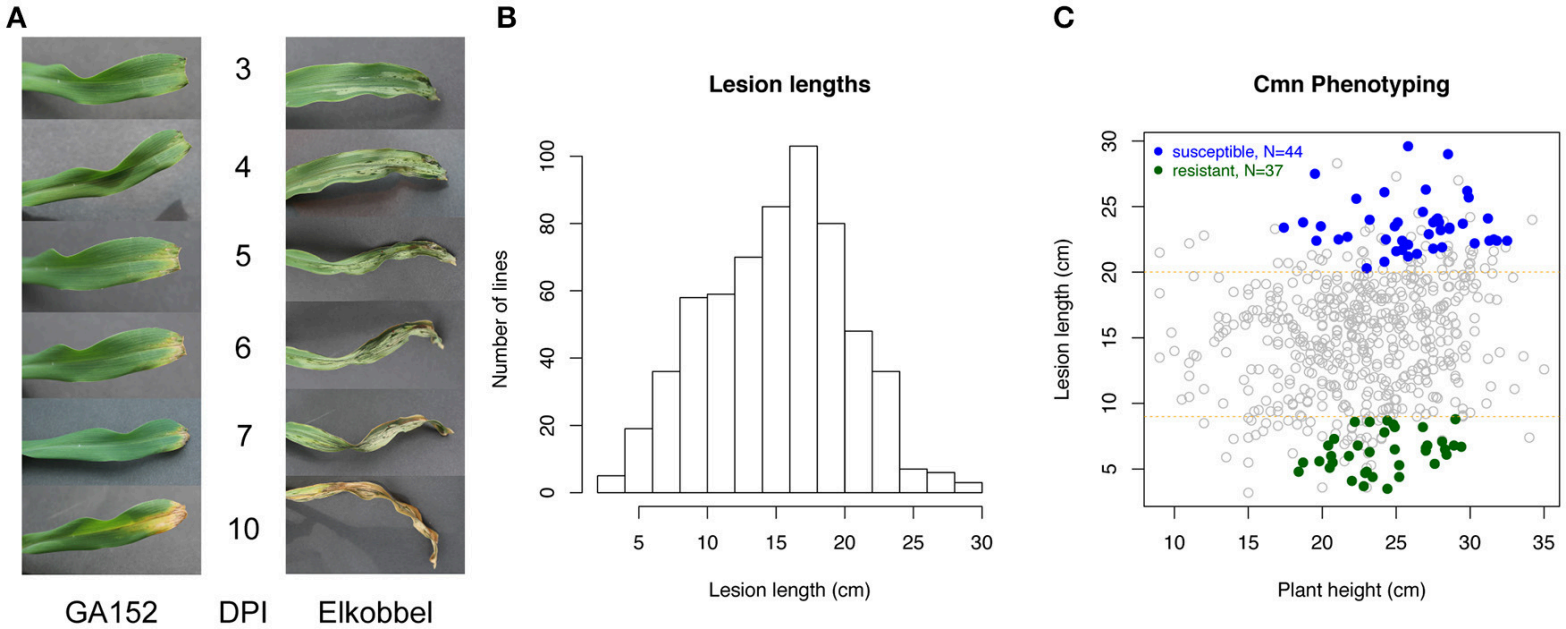
Quantification of lesions caused by a Cmn strain. **(A)** The lesion development on third leaf of three-leaf seedlings of a resistant maize line (GA152) and a susceptible maize line (Elkobbel). **(B)** Histogram of lesion lengths of 615 maize lines at 13 DPI. **(C)** Selection of highly resistant (R) and highly susceptible (S) maize lines based on the best linear unbiased estimation (BLUE) values of lesion lengths.

To establish a rapid and reproducible method for quantification of GW, the effects of growth stages and leaf positions on lesion expansion were first tested on seedlings of a maize inbred line Mo17. The results indicated that three-leaf seedlings overall exhibited longer lesion expansion compared to four-leaf seedlings in the same time period, allowing for greater potential variation in symptoms (Supplementary Figure 2A). Three-leaf seedlings of 25 maize inbred lines were inoculated on the second or third leaf, and the lesion lengths were measured every 2 days from 3 to 13 DPI. Two-way ANOVA analysis on the factors of inoculation leaf and maize genotype found the greater mean expansion of lesions on third leaves vs. second leaves of diverse maize lines. Also high variation in lesion length between maize lines and low variation within individuals of a given line were observed on the third leaf compared to the second leaf (Supplementary Figure 2B), suggesting that the third leaf at the three-leaf seedling stage is optimal for lesion phenotyping. Twenty-four NAM parents and Mo17 showed a wide range of among-genotype variation in lesion length using this phenotyping method at 13 DPI (Supplementary Figure 3). The method was used to measure lesion lengths at 13 DPI to represent host resistance levels for 615 maize lines. Overall, maize lines showed a wide range of variation in lesion length from 2.8 to 32.1 cm at 13 DPI (Figures 1A,B, Supplementary Table 2; a time-lapse video of a highly susceptible line provided in Supplementary Movie 1). To account for potential variation from plant growth habits of the individual lines, a linear regression model was fitted for lesion lengths based on plant heights. Based on corrected lesion lengths, 37 so-called highly resistant (R) genotypes and 44 highly susceptible (S) genotypes were selected from 615 lines as extreme phenotypes (XP) (see Methods) (Figure 1C).

### Differential gene copy number in R and S pools

Two biologically replicated pools of R lines and pools S lines selected from 615 lines were subjected to whole genome shotgun (WGS) sequencing. In total, ~50.3x (125.7 Gb) and 49.6x (123.9 Gb) sequences were generated for the R and S pools, respectively. WGS sequencing data were aligned to the B73 reference genome and uniquely mapped reads were used for determining read depths per gene. We employed a novel approach, XP-CNV, to examine CNV between the R and S pools through comparing genic read depths. Each of the genes (*N* = 37,483) with at least 20 total reads from all the four samples (two biological replicates of R and S pools) was subjected to a statistical test for the null hypothesis of no difference in sequencing depths between the R and S pools (Love et al., 2014). As a result, 141 genes exhibited significantly differential read depths between R and S pools. These genes were considered as candidate CNV genes showing distinct copies in the R and S lines. Among the candidate CNV genes, 90 and 51 genes were displayed more copies (Up-CNV) and fewer copies (Dn-CNV) in R vs. S, respectively (Supplementary Table 3).

**Figure 2:**
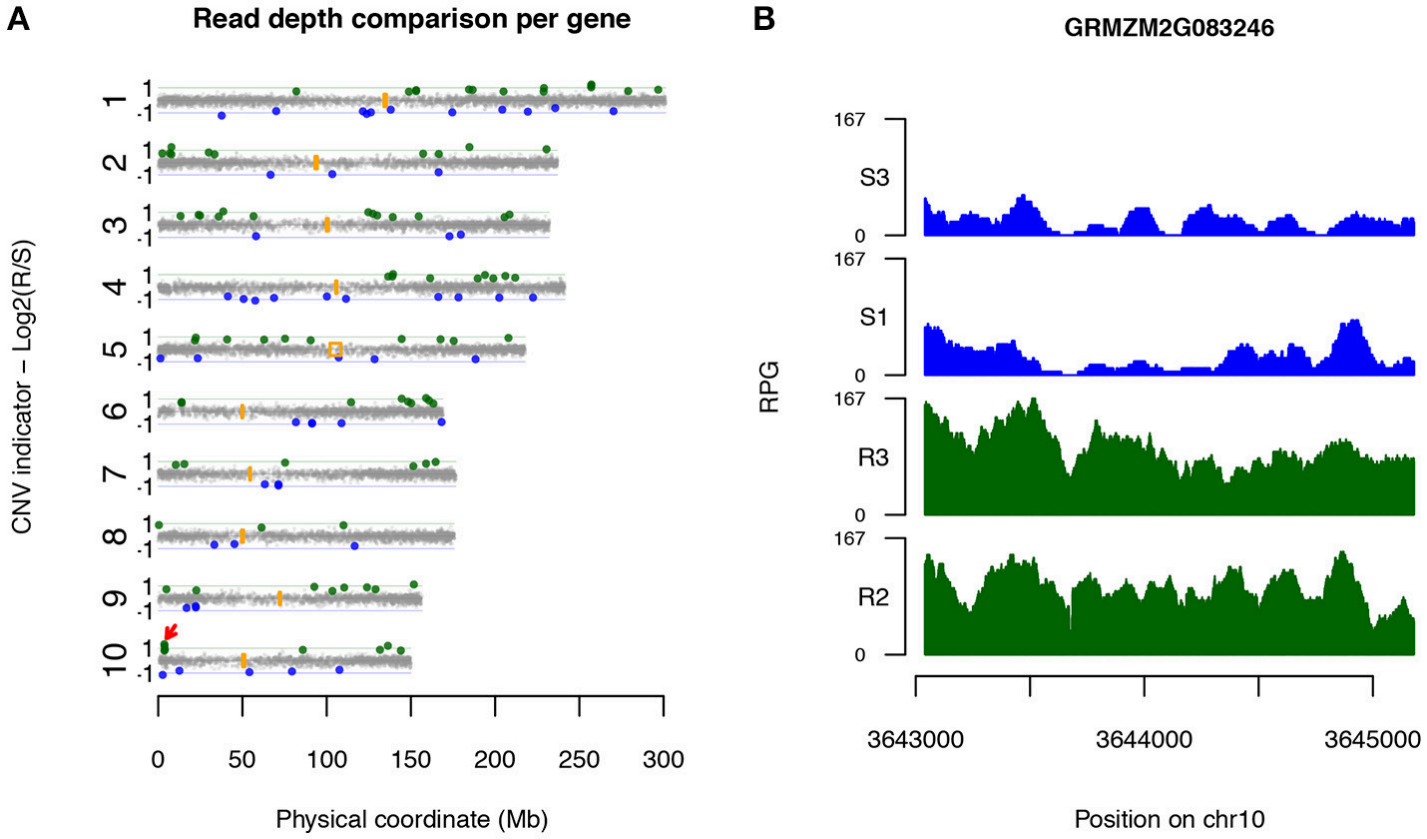
Candidate disease-associated genes identified *via* XP-CNV. **(A)** A genome-wide view of the distribution of sequencing depth comparison between highly R pools and highly S pools across 10 maize chromosomes. Each dot designates a gene. Y-axis represents log2 values of ratios of read depths of R to S, signifying CNV between R and S pools. Genes with statistically significantly higher and lower read depths of R vs. S pools were interpreted as Up-CNV (green) and Dn-CNV (blue), respectively. Orange boxes indicate centromeric positions. Red arrow points to the *rp1* locus. **(B)** Read depths (reads per gigabit total aligned reads, RPG) across the Up-CNV gene GRMZM2G083246 from the pools of two highly R (R2 and R3) and two highly S (S1 and S3) pools.

Candidate CNV genes were found on all 10 maize chromosomes (Figure 2A). Some genes were physically close. Six loci on chromosomes 1, 2, 6, and 10, each of which harbors multiple Up-CNV genes in relation to R pools, were closely clustered within 250 kb of each other, while four loci were observed close together on chromosomes 2, 6, and 7 for Dn-CNV genes in relation to R pools. Noticeably, a locus at the short arm on chromosome 10 contained four Up-CNV genes (GRMZM2G083246, GRMZM2G349565, GRMZM2G005134, and GRMZM2G143769). Functional annotation revealed that all these four genes are *rp1* family members. The *rp1* gene, GRMZM2G083246, displayed the highest fold change (2.5x) of sequencing depths in R pools vs. S pools among all Up-CNV genes (Figure 2B). *Rp1* is an NLR gene that was previously discovered to confer race-specific resistance to common rust of maize (Smith et al., 2004).

Besides *rp1*, a number of other Up-CNV genes were associated with plant disease responses. For example, a *SEC-14*-related gene (GRMZM2G363377), and one of top Up-CNV genes, displayed a 2.4x fold change in sequencing depths of R pools vs. S pools (Table 1). *SEC-14*-like genes encode phospholipid transfer proteins, which can play an important role in plant innate immune responses (Kiba et al., 2014). Another Up-CNV gene (GRMZM2G362303) encodes a wall-associated kinase (WAK). WAK family members have been demonstrated to be associated with plant defenses, including the rice OsWAK1, *Arabidopsis* WAK10 and WAK22 (Diener and Ausubel, 2005; Li et al., 2009b; Meier et al., 2010), and maize ZmWAK (Zuo et al., 2015). Gene ontology (GO) enrichment analysis of Up-CNV vs. Dn-CNV showed that the GO term transport (GO:0006810) was overrepresented in Dn-CNV (*p* < 0.03). Other GO terms associated with protein kinase, electron carrier, ion binding and heme binding, although not significantly overrepresented, occurred at least three times in Up-CNV but were absent in Dn-CNV.

**Table 1:** Information of the top ten Up-CNV and the top 10 Dn-CNV genes.

**GeneID**	**log2FC^a^**	***p*-Value**	**padj^b^**	**Chr**	**Start**	**End**	**Description^c^**
GRMZM2G083246	1.32	4.05E-09	2.54E-05	10	3643040	3645180	LRR and NB-ARC domains containing disease resistance protein (*rp1*)
GRMZM2G026176	1.28	1.51E-09	1.42E-05	1	257112452	257127180	Unknown
GRMZM2G363377	1.25	5.04E-11	9.48E-07	2	184717427	184745555	Sec14p-like phosphatidylinositol transfer family protein
GRMZM2G169013	1.25	2.08E-08	1.12E-04	2	7859326	7861362	Unknown
GRMZM2G702514	1.19	7.19E-12	2.70E-07	10	136325395	136339339	Flavanone 3-hydroxylase
GRMZM2G357081	1.19	1.21E-09	1.42E-05	1	257033199	257043603	Shaggy-like kinase 13
GRMZM2G349565	1.17	6.10E-07	1.76E-03	10	3764373	3766967	LRR and NB-ARC domains containing disease resistance protein (*rp1*)
GRMZM2G009770	1.12	9.94E-07	2.44E-03	9	151759076	151762771	Leucine-rich repeat protein kinase family protein
GRMZM2G127619	1.08	1.16E-06	2.57E-03	6	159004500	159005754	Unknown
GRMZM2G701441	1.08	2.77E-09	2.08E-05	3	38609811	38632417	Unknown
GRMZM2G086935	−0.94	3.38E-05	2.70E-02	7	71388331	71391082	Unknown
GRMZM2G106165	−0.95	2.06E-05	1.94E-02	4	50720961	50724861	Serine-type carboxypeptidase
GRMZM2G049027	−0.96	5.95E-05	3.82E-02	6	91353470	91355242	Cleavage and polyadenylation specificity factor 30
GRMZM2G575323	−0.96	4.89E-05	3.47E-02	1	126269847	126270723	Unknown
GRMZM2G164672	−0.97	3.74E-05	2.87E-02	1	174565242	174567097	Unknown
GRMZM5G829946	−0.98	2.30E-05	2.06E-02	2	66708324	66712872	PLC-like phosphodiesterases superfamily protein
GRMZM2G166695	−1.08	6.98E-08	2.92E-04	4	57590015	57595523	Remorin family protein
GRMZM2G009624	−1.09	1.04E-06	2.44E-03	1	123774374	123777663	RNA-binding (RRM/RBD/RNP motifs) family protein
GRMZM2G320305	−1.15	8.25E-08	3.10E-04	10	2690434	2694516	Ferredoxin-NADP(+)-oxidoreductase 2
GRMZM2G014323	−1.22	3.94E-08	1.85E-04	1	37667524	37685753	Cell division control 2

a *Log2-fold change in read depth between R and S pools*.

b *Adjusted p-values*.

c *Gene functional annotation from Phytozome or maizesequence.org*.

### Confirmation of the association of the *rp1* locus with GW resistance

**Figure 3:**
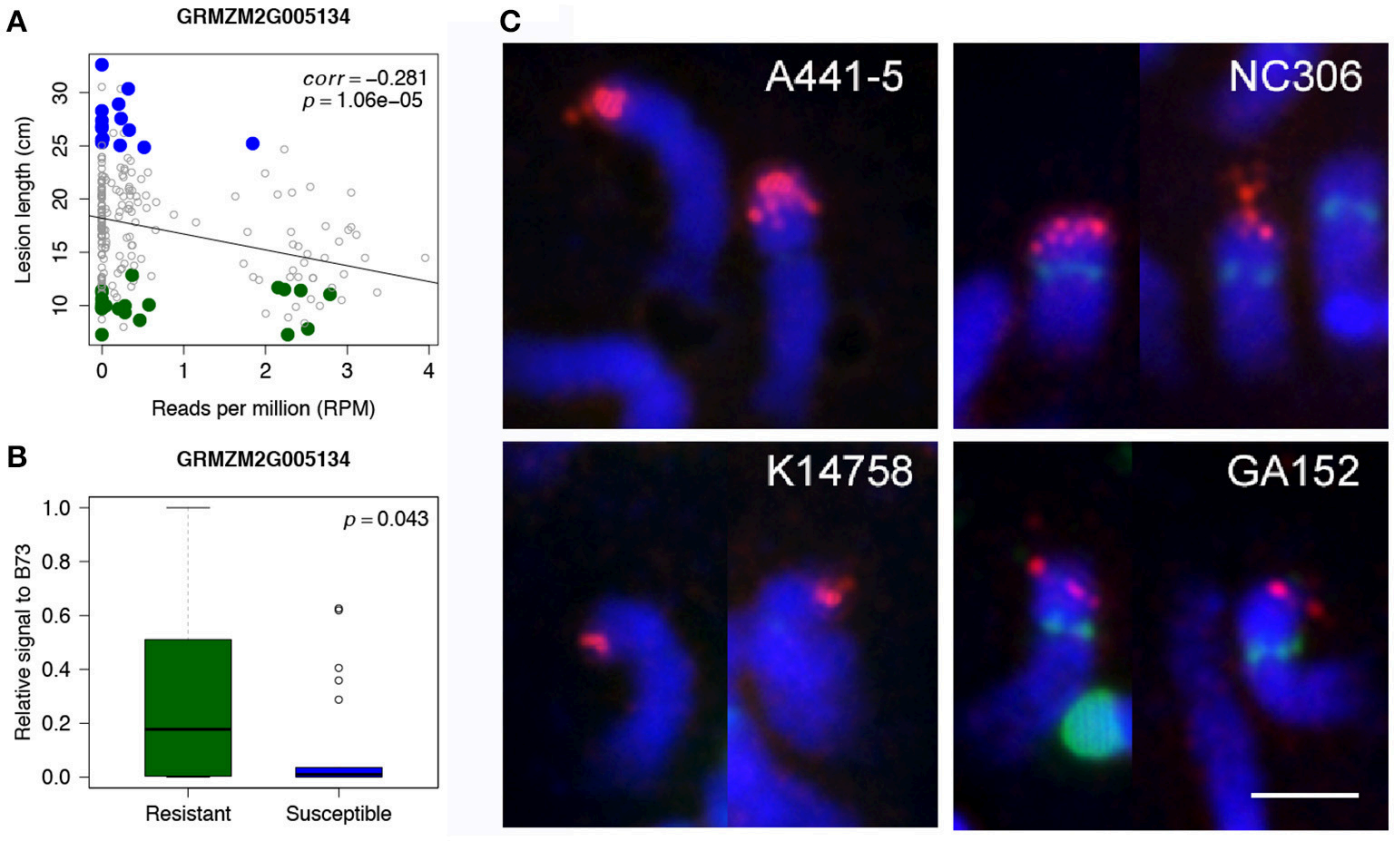
Association of an *rp1* gene with disease resistance. **(A)** Correlations between read depths of an *rp1* gene (GRMZM2G005134) and lesion lengths of 239 lines from the Maize 282 Association Panel. Highly resistant and susceptible lines that are overlapped with the Maize 282 Association Panel are labeled in green and blue, respectively. **(B)** Boxplots of qPCR signals of GRMZM2G005134 in the group of selected R lines and B73 as well as the group of S lines. **(C)** FISH of the 1.7kb *rp1* probe on somatic late prophase of chromosome 10 of four maize lines. *Rp1* signals are in red; CentC, NOR, and 5S rDNA repeats are in green; chromosomes counterstained with DAPI are in blue. Bar corresponds to 5 μm.

WGS data of individual lines of 239 maize accessions from the Maize 282 Association Panel was used to corroborate the association of *rp1* with disease resistance. The 239 lines are a subset of 615 lines whose disease resistance levels (lesion lengths) were phenotyped in this study. For each *rp1* gene, the correlation of lesion lengths with normalized read counts of the gene were examined. All four *rp1* genes identified as Up-CNV genes showed negative correlations with susceptibility as determined by lesion length (all four *p*-values smaller than 0.002, correlations were from −0.346 to −0.203) (Figure 3A, Supplementary Figure 4). Quantitative PCR (qPCR) of one of *rp1* genes, GRMZM2G005134, on B73, 22 R lines and 22 S lines, which randomly selected from highly R and S lines, using a specific primer pair of GRMZM2G005134, showed a high correlation between qPCR signals and sequencing depths of this gene (correlation = 0.831, *p* = 3.04e-6) (Supplementary Figure 5). Of 22 R lines and 22 S lines, 11 R and 17 S lines displayed extremely low qPCR signals, indicating that GRMZM2G005134 probably was absent in these lines (Supplementary Figure 6). The *t*-test comparing the R lines and B73 with S lines resulted in a significant association between qPCR signals of GRMZM2G005134 and GW resistance (*p* < 0.05) (Figure 3B). Eight R and S lines were randomly selected for fluorescent *in situ* hybridization (FISH) using a 1.7kb *rp1* probe to examine the overall *rp1* FISH signals (Figure 3C, Supplementary Figure 7). Resistant and susceptible lines showed strong or weak *rp1* FISH signals, depending on the line. For examples, A441-5, a susceptible line, and NC306, a resistant line, had strong *rp1* signals, whereas K14758, a susceptible line, and GA152, a resistant line had weak *rp1* signals (Figure 3C). Thus, if *rp1* is responsible for the resistance, specific combinations or specific *rp1* paralogs rather than the total number of *rp1* paralogs are associated with resistance.

**Figure 4:**
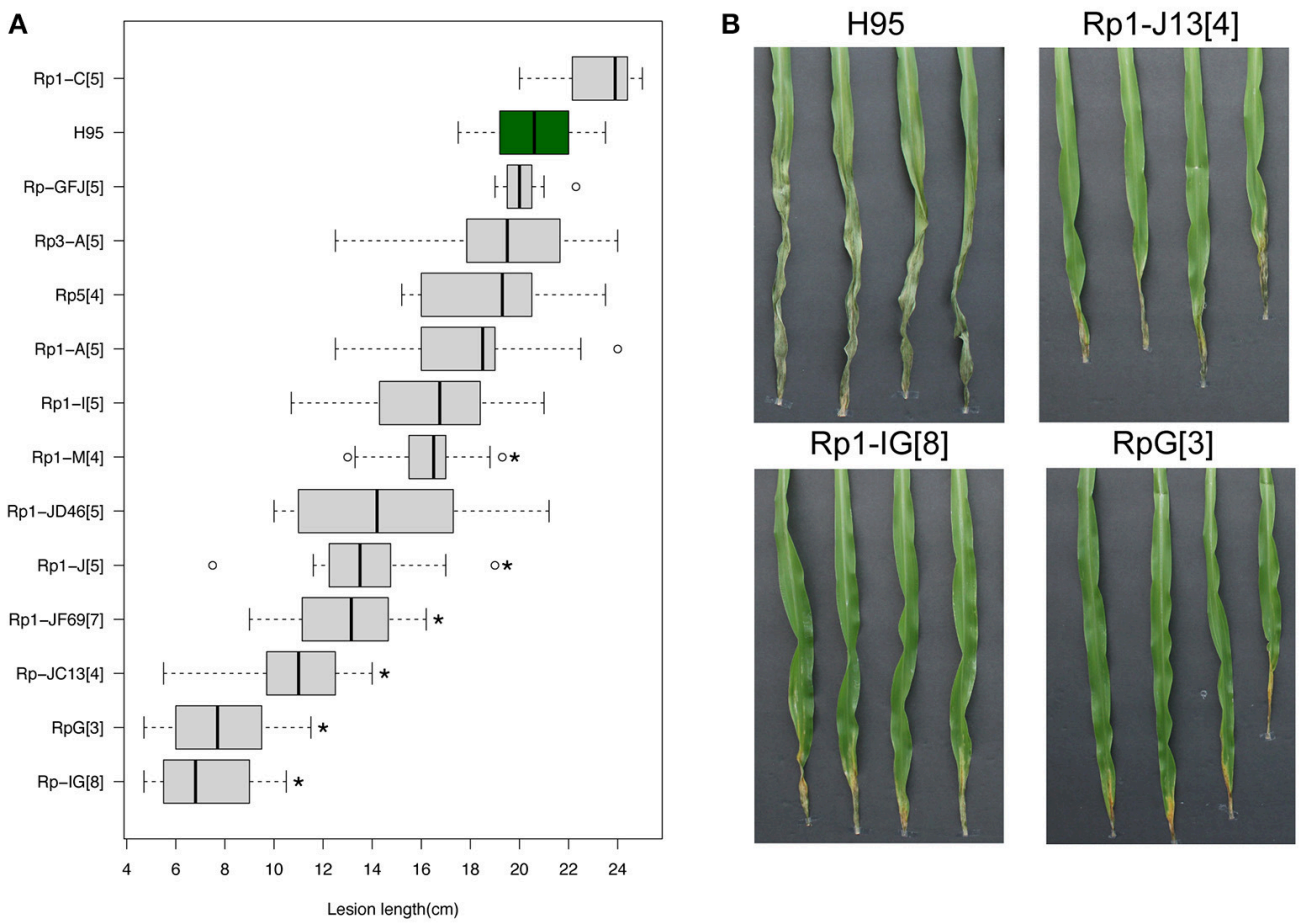
Effects of multiple *Rp1* accessions on disease resistance. **(A)** Distribution of lesion lengths at 13 DPIs in multiple *Rp1* accessions of which the *rp1* locus was introgressed into the inbred line H95. In the boxplot, the vertical line within each box indicates a median of lesion lengths. Asterisks indicate statistically significant differences between H95 and an *Rp1* accession (*t*-test, ^*^p < 0.001). **(B)** Goss's wilt phenotype of H95 and three *Rp1* accessions, *RpG* (an *Rp1* haplotype), *Rp1-IG*, and *Rp1-JC13*, at 13 DPIs with a Cmn strain. Numbers in square brackets indicate the times of backcross generations.

Multiple *Rp1* haplotypes, containing various *rp1* copies and different *rp1* genes (Collins et al., 1999), were collected and introgressed to an inbred line H95 that is highly susceptible to both common rust and GW. All *Rp1* haplotypes confer race-specific resistance to common rust. We phenotyped the introgression *Rp1* lines to assess GW resistance. As additional controls, *Rp3* and *Rp5* introgression lines were also tested. Of eleven *Rp1* accessions, three lines, containing *RpG* (an *rp1* haplotype), *Rp1-IG* or *Rp1-JC13*, showed high levels of resistance in comparison to H95, with 61.6% (*p* = 2.4e-6), 64.9% (*p* = 1.4e-11), and 47.5% (*p* = 2.8e-10) reduction in lesion length, respectively (Figure 4A). All three lines showed necrotic and/or chlorotic symptoms at inoculation sites, resembling an HR reaction (Figure 4B). Some *Rp1* accessions exhibited moderate resistance to GW in comparison to H95, including accessions *Rp1-JF69, Rp1-J*, and *Rp1-JD46* with 36.8% (*p* = 3.2e-8), 33.8% (*p* = 7.3e-6), and 29.4% (*p* = 1.2e-3) reduction in lesion length, respectively. Both *Rp3* and *Rp5* accessions did not show enhanced resistance. Quantification of disease resistance of these *rp1* haplotypes corroborated the association between the *rp1* locus and GW resistance.

## Discussion

The genetic basis of host/pathogen interactions to *Clavibacter*, in any system, is poorly understood. This study examined the genetic basis of the emerging maize Gram-positive bacterial disease GW. We applied XP-CNV to identify GW-associated CNV and discovered an associated locus in the region of the R locus *rp1* to *Puccinia sorghi*, the causal agent of common leaf rust (Hulbert, 1997; Smith et al., 2004). The co-localization was corroborated by the observation of effective resistance of multiple but not all *Rp1* accessions. Resistance to rust and possibly CMN is due to either the unique combination of *rp1* genes (Hu et al., 1997) or intragenic recombination events generating novel *rp1* genes at the *rp1* locus (Smith et al., 2010). Some of these haplotypes mediate spontaneous defense (lesion mimic) responses even in the absence of rust infection (Hu et al., 1997; Chintamanani et al., 2010; Smith et al., 2010). Lesion mimic phenotypes have not been observed in most of the GW resistant lines identified in this study. The GW resistance identified in these lines is thus probably due to a specific response to the bacterial pathogen and not a non-specific induction of defense responses. In the simplest application of the results, manipulation of known resistant *rp1* haplotypes in elite germplasm may provide enhanced resistance to GW.

Many plant disease resistance genes are dispensable to the general fitness of the plant (Collins et al., 2003; McHale et al., 2012; Yao et al., 2015; Hardigan et al., 2016). Therefore, in theory, some CNV such as PAV or tandem duplication should be detectable by SNP markers that exhibit high linkage disequilibrium with CNV locations. A human study indicated that 77% of CNVs were effectively tagged by SNPs, suggesting GWAS can capture the majority of CNV (Conrad et al., 2010). Genotyping by CNV of multiple unlinked homologous family members may obfuscate SNP analysis. Unlike SNPs or small insertions or deletions (InDels), PAV or other CNV have not been well established. In this study, XP-CNV was used with bulked WGS sequencing data for the purpose of reducing sequencing cost. Sequencing depths per gene were determined to infer average gene copy numbers of individuals in each GW R and S pools. The statistical comparison between the R and S pools resulted in a set of genes with differential average gene CNVs between the two groups, and many Up-CNV genes in R pools seemed to be defense-related. The strategy of XP-CNV should be efficient for direct identification of causal CNV genes.

We have utilized sequencing data of the Maize 282 Association Panel to verify the GW association with *rp1* genes, suggesting that the XP-CNV result is largely repeatable. However, XP-CNV analyses are subject to false discovery. First, organelle sequences homologous to nuclear chromosomes could result in high variation in read depths (Lough et al., 2015). The number of organelles, such as mitochondria or chloroplasts, varies in different tissues and the percentage of organelle genomes in DNA samples is subject to DNA extraction procedures. Therefore, falsely discovered CNV can result from organelle sequences. Here, two biological replicates of each R and S pool were done to minimize the effect of organelle variation. Second, the method is influenced by the population structure of selected lines, which could lead to spurious association (Balding, 2006). One solution is to generate sequences of individual lines that can be used to assess population structure, and to establish phenotype-CNV association with the control of population structure.

Genomic structural variation (SV) includes CNV and other SV types such as inversions and rearrangements, and only CNV was examined in this study. It is still challenging to reliably genotype SV other than CNV in a population scale. However, the advance of longer read sequencing and bioinformatics algorithms has improved the calling accuracy and reliability (Pirooznia et al., 2015; Dong et al., 2016; Huddleston et al., 2016; Peng et al., 2016). In future, with the continuous decrease of sequencing cost and improvement of sequencing technologies, the majority of plant germplasms will be sequenced in a decent depth. Phenotypic association of CNV and perhaps other genomic structural variation would be a regular analysis in addition to regular GWAS.

Through XP-CNV, we identified the association between the *rp1* genes and disease resistance to GW caused by the Gram-positive bacterium Cmn. HR-like resistant responses were observed for multiple *Rp1* haplotypes. It is unknown whether one or multiple separate genes with separate elicitors occur at the locus. The *Rp1* locus encodes a number of nucleotide binding site/leucine rich repeat proteins, which are known to participate in the recognition of pathogen effectors (Deyoung and Innes, 2006). In Gram-negative bacteria, many of the known effectors are delivered to plant cells through the type III systems (T3SS). However, no secretion systems equivalent to T3SS has been identified in Cmn (Bentley et al., 2008; Gartemann et al., 2008; Lu et al., 2015b). The known virulence factors of related *Clavibacter* pathogens are secreted by the type II secretion system (T2SS). Alternatively, toxins or other non-proteinaceous compounds may trigger the HR. The revelation of the association between the *rp1* locus and GW provides a system to elucidate molecular interactions between hosts and bacterial species of *C. michiganensis*.

## Author contributions

YH, JR, FW, AR, and SL designed experiments; YH, HW, JF, and SL analyzed data. YH, JR, ZP, AU, HL, and TD performed wet-lab experiments; SH maintained and provided *rp1* lines; YH, FW, and SL wrote the manuscript with the input from ZP, AR, TD, JF, SH, and HW. All authors read and approved the final manuscript.

### Conflict of interest statement

The authors declare that the research was conducted in the absence of any commercial or financial relationships that could be construed as a potential conflict of interest. The reviewer KM and handling Editor declared their shared affiliation.
