# Supplementary material for: Analysis of Extreme Phenotype Bulk Copy Number Variation (XP-CNV) Identified the Association of rp1 with Resistance to Goss's Wilt of Maize
Source: Front Plant Sci. 2018 Feb 9;9:110. doi: 10.3389/fpls.2018.00110 (PMC5812337; doi:10.3389/fpls.2018.00110)
Supplement: Supplementary file 4 [file DataSheet1.docx]

Supplementary Material

**Extreme phenotype bulk copy number variation (XP-CNV) analysis facilitates the genetic dissection of host resistance to Goss’s wilt of maize**

Ying Hu, Jie Ren, Zhao Peng, Arnoldo Alvarez Umana, Ha Le, Tatiana Danilova, Junjie Fu, Haiyan Wang, Alison Robertson, Scot H. Hulbert, Frank F. White, Sanzhen Liu^*^

*** Correspondence:** Corresponding Author: liu3zhen@ksu.edu

**Supplementary Table 1. Primers for qPCR**

| Primer | Primer sequence |
| --- | --- |
| Gene specific primer pair (GRMZM2G005134) | Forward: ATGGTCCCACCAAATCTTGA |
|  | Reverse: ATGCTATGTTGGGGCAAAAC |
| Control primer pair (GRMZM2G126010) | Forward: GATGATGCGCCAAGAGCTG |
|  | Reverse: GCCTCATCACCTACGTAGGCAT |

**Supplementary Table 2. Phenotype data of 615 maize lines** (see the separated supplemental file).

**Supplementary Table 3. List of significant Up-CNV and Dn-CNV genes** (see the separated supplemental file).


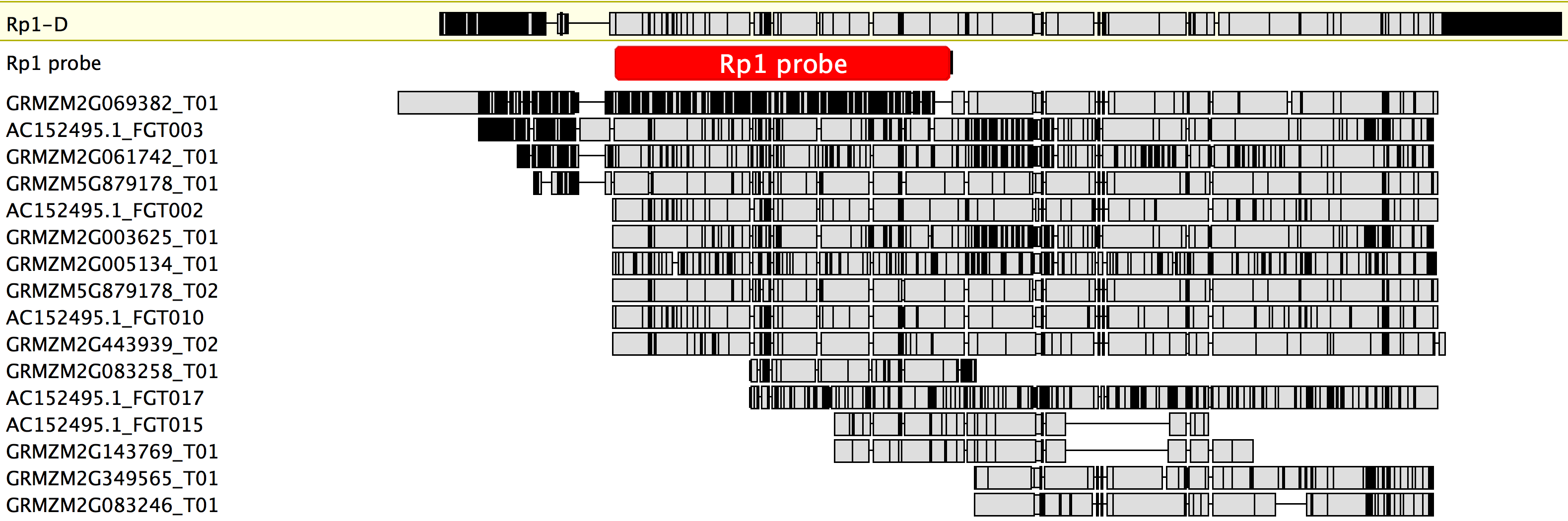


**Supplementary Figure 1. DNA Sequence alignments of 16 *rp1* transcripts identified in B73 (Ref v2) with *Rp1-D*.** Gray indicates region where sequences of *rp1* homologs are identical to *Rp1-D* gene. Black highlights polymorphisms of *rp1* sequences in comparison to the *Rp1-D* sequence. The sequence of the *rp1* probe used for FISH experiment was highlighted in red.


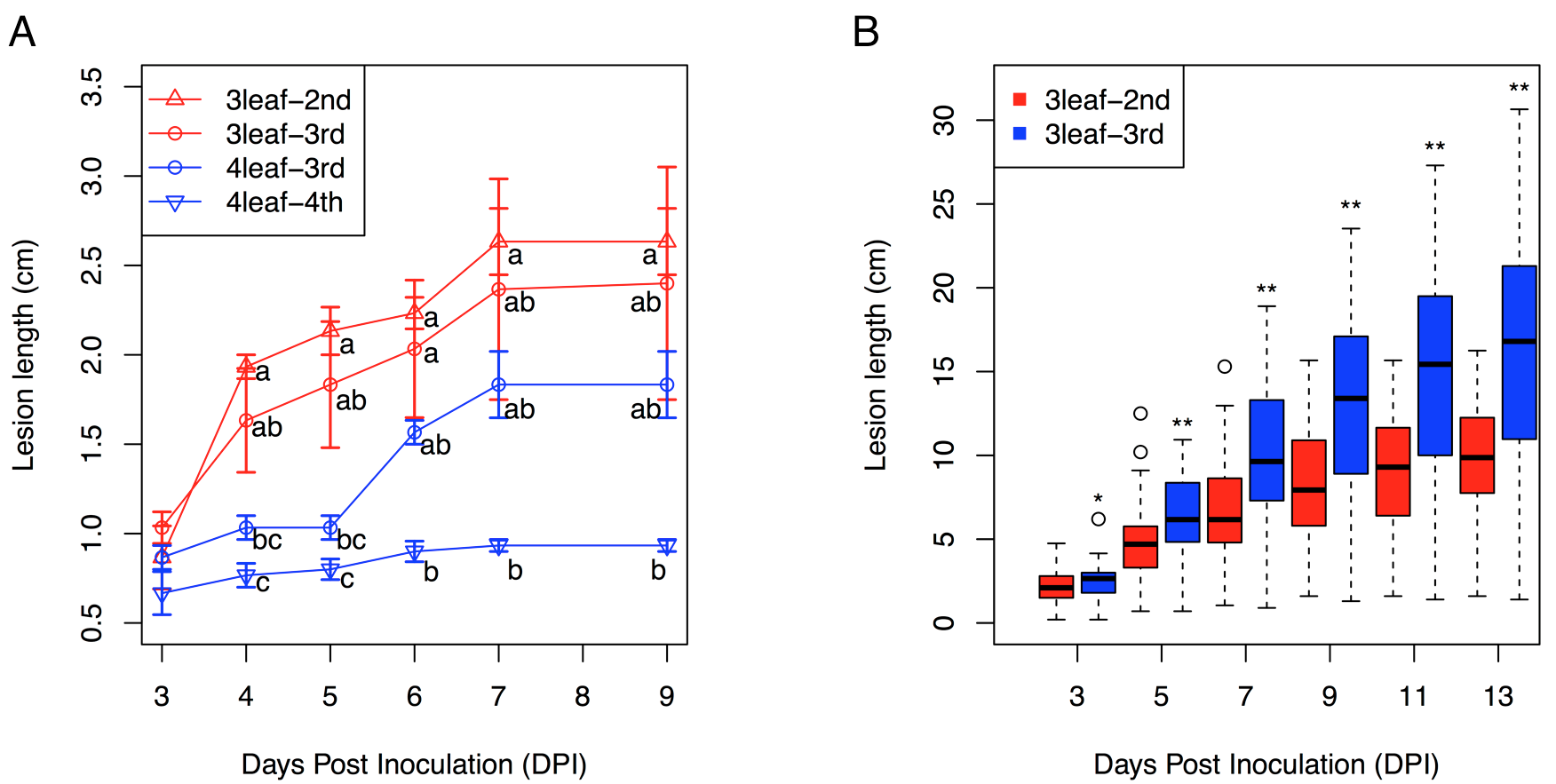


**Supplementary Figure 2. Leaf lesion lengths of different maize lines inoculated with Cmn06-1.** (**A**) Leaf lesions were measured on second (red triangle up) and third leaf (red circle) on 3-leaf-stage Mo17 seedlings, and third (blue circle) and forth leaf (blue triangle down) on 4-leaf-stage of Mo17 seedlings. Error bars represent the means ± SE (standard error) (n = 4). Tukey’s honestly significant difference test (Tukey’s HSD) was performed for each DPI. Groups were labeled if significant mean differences between groups were identified. (**B**) Boxplots of leaf lesion length measured on second (red) and third leaves (blue) of 25 different 3-leaf-stage maize lines. Circles on top of some boxplots are the outliers outside 1.5 times of the difference between the third quartile and the first quartile beyond the third quartile. Data were collected every two days from 3 to 13 days post inoculation (DPI), representing a time course of the response of different leaf stages of maize lines to infection with Cmn06-1. Two-way ANOVA considering two factors, inoculation leaf and genotype, was performed at each DPI. The significance levels of mean difference in lesion length between second leaves and third leaves were labeled with asterisk (* p-value < 0.01, ** p-values < 0.001).


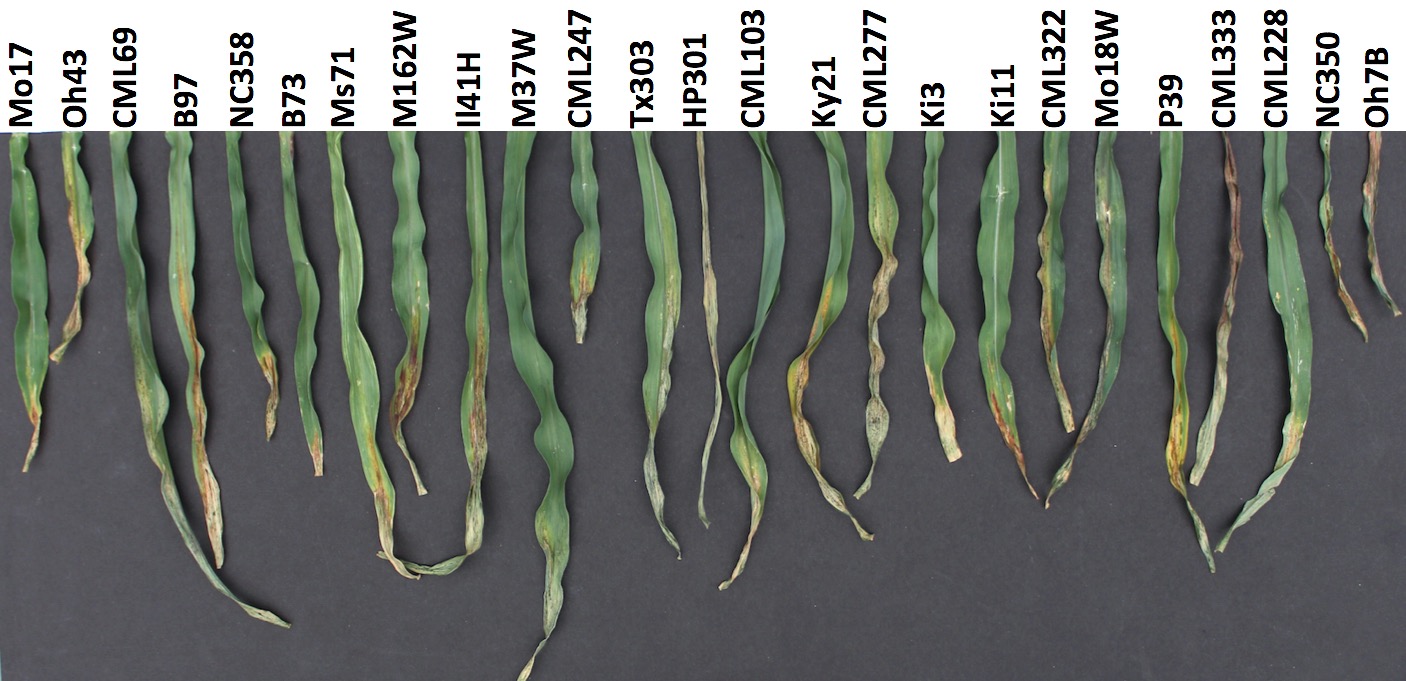


**Supplementary Figure 3.** **Quantification of lesions caused by Cmn06-1 in 24 NAM founders and Mo17.** Three-leaf-stage seedlings were inoculated with Cmn06-1 and images were taken at 13 DPIs.

**Supplementary Figure 4. Correlations between normalized read counts and phenotypic values for three *rp1* paralogs.** The highly R and S lines were labeled in dark green and blue.

**Supplementary Figure 5. Correlation between qPCR signals of and normalized read counts data of GRMZM2G005134.** The R and S lines were labeled in green and blue, respectively.

**Supplementary Figure 6. qPCR signal of GRMZM2G005134 in highly resistant and susceptible lines.** Data are expressed as relative values based on B73 as reference sample set to 1.0. Error bars represent the means ± SD (*n* = 3). The highly resistant and susceptible lines were labeled in dark green and blue. B73 was labeled in green.


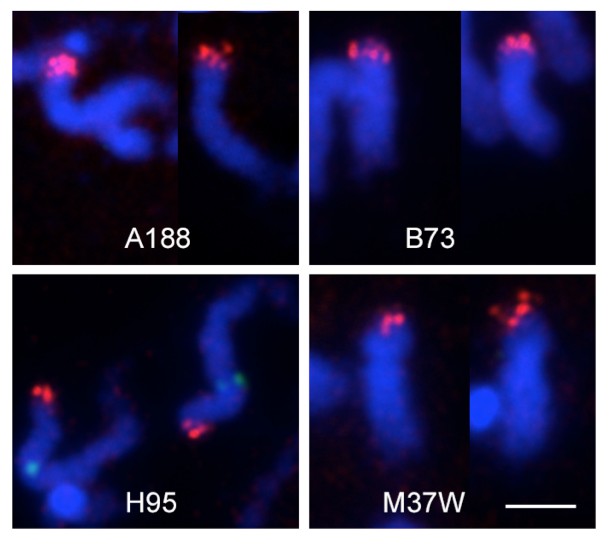


**Supplementary Figure 7. FISH of the 1.7kb *rp1* probe on somatic late prophase of chromosome 10 of four maize lines.** *Rp1* signals are in red; CentC, NOR and 5S rDNA repeats are in green; chromosomes counterstained with DAPI are in blue. Bar corresponds to 5 μm.

**Supplementary Movie 1. Time-lapse animation of disease development on A188 leaf.** Photos were taken with a Raspberry Pi every 12 minutes during days. The animation of a maize inbred line A188 displayed the initiation and the extension of water soaking, and gradual wilting of inoculated leaves. (see the separated supplemental file)
